# Potential Involvement of miR-144 in the Regulation of Hair Follicle Development and Cycle Through Interaction with *Lhx2*

**DOI:** 10.3390/genes15111454

**Published:** 2024-11-11

**Authors:** Guangxian Zhou, Xiaolong Wang, Yulin Chen, Danju Kang

**Affiliations:** 1 College of Coastal Agricultural Sciences, Guangdong Ocean University, Zhanjiang 524088, China; zhougx@gdou.edu.cn; 2 College of Animal Science and Technology, Northwest A&F University, Yangling 712100, China; xiaolong.wang@vip.163.com (X.W.); chenyulin@nwsuaf.edu.cn (Y.C.)

**Keywords:** *Lhx2*, miR-144, hair follicle, hair growth and development, hair follicle cycle

## Abstract

Background: Cashmere, known as “soft gold”, is a highly prized fiber from Cashmere goats, produced by secondary hair follicles. Dermal papilla cells, located at the base of these follicles, regulate the proliferation and differentiation of hair matrix cells, which are essential for hair growth and cashmere formation. Recent studies emphasize the role of microRNAs (miRNAs) in controlling gene expression within these processes. Methods: This study centered on exploring the targeted regulatory interaction between miR-144 and the *Lhx2* gene. Utilizing methodologies like miRNA target prediction, luciferase reporter assays, and quantitative PCR, they assessed the interplay between miR-144 and Lhx2. Dermal papilla cells derived from Cashmere goats were cultured and transfected with either miR-144 mimics or inhibitors to observe the subsequent effects on Lhx2 expression. Results: The results demonstrated that miR-144 directly targets the *Lhx2* gene by binding to its mRNA, leading to a decrease in Lhx2 expression. This modulation of Lhx2 levels influenced the behavior of dermal papilla cells, affecting their ability to regulate hair matrix cell proliferation and differentiation. Consequently, the manipulation of miR-144 levels had a significant impact on the growth cycle of cashmere wool. Conclusions: The findings suggest miR-144 regulates hair follicle dynamics by targeting *Lhx2*, offering insights into hair growth mechanisms. This could lead to innovations in enhancing cashmere production, fleece quality, and addressing hair growth disorders. Future research may focus on adjusting miR-144 levels to optimize Lhx2 expression and promote hair follicle activity.

## 1. Introduction

Dermal papilla cells (DPCs), fibroblast-like cells originating from the dermis, intricately govern various aspects of hair follicle development and growth, including the phases of regression and regeneration [1,2,3]. Functioning as critical components within the complex hair follicle niche, DPCs play a pivotal role in orchestrating the proliferation, differentiation, and apoptosis of hair matrix cells [2,4,5]. Despite advancements, the signals emitted by DPCs that trigger changes in the hair matrix during regeneration remain a subject of ongoing investigation. Therefore, the identification of key genes and an exploration into the molecular mechanisms underlying hair follicle regression or regeneration is of paramount importance for advancing our understanding of this intricate process.

MicroRNAs (miRNAs) represent a class of non-coding RNAs intricately involved in the post-transcriptional regulation of genes by targeting and binding to the mRNAs of specific genes [6,7]. Their regulatory functions hold paramount importance in the intricate processes of hair follicle development, cyclic changes, and cell proliferation/differentiation [8,9,10,11]. Numerous studies have elucidated the involvement of several miRNAs in orchestrating the growth of hair follicles. Among these, microRNA-144 (miR-144) has emerged as a significant player, with its expression in the telogen phase exhibiting variations across different growth stages of hair follicles [12]. Previous research has highlighted the pivotal role of miR-144 in the differentiation and proliferation of mesenchymal stem cells [13,14]. However, the molecular intricacies governing how miR-144 precisely regulates the hair follicle growth cycle remain enigmatic. Despite its recognized importance, the specific mechanisms through which miR-144 exerts its influence on the dynamic processes of hair follicle development and cyclic changes are yet to be fully understood. Further exploration into these molecular intricacies promises to unravel novel insights into the regulatory network orchestrating the hair follicle growth cycle.

LIM/homeobox protein 2 (*Lhx2*), a distinguished member of the expansive protein family characterized by the LIM domain and a distinctive cysteine-rich zinc-binding domain, intricately governs pivotal developmental processes [15]. The absence of *Lhx2* impedes fetal wound healing in mice and disrupts the intricate process of hair follicle development [16]. Notably, *Lhx2* serves to sustain stem cell characteristics within hair follicles, and the absence of *Lhx2* results in an inability to maintain the quiescent state, leading to premature activation [17]. Conversely, conflicting studies suggest that *Lhx2* plays a role in both the generation and regeneration of hair. Transgenic mice with heightened *Lhx2* expression fail to induce the anagen phase [18]. However, the precise role and molecular mechanisms of *Lhx2* in dermal papilla cells (DPCs) remain inadequately elucidated.

In the current study, we observed elevated expression of miR-144 in skin tissue during the telogen phase, and further investigation revealed that miR-144 suppressed the translation of *Lhx2* mRNA. Additionally, we demonstrated the regulatory impact of miR-144 on the expression of key genes (*Lhx2*, *Sox9*, *Lgr5*, etc.) in DPCs. Collectively, these findings suggest that miR-144 holds the potential to intricately regulate the development and cyclic processes of hair follicles.

## 2. Materials and Methods

### 2.1. Goat Skin Sample Collection

Skin tissue samples of three two-year-old female Shanbei Cashmere goats were procured from a goat farm situated in the Hengshan District of Yulin, China (located at 37°21′~38°14′ N and 108°56′~110°01′ E). The sampling location of the goat skin was at a point 5 cm away from the dorsal midline between the 12th and 13th ribs. The sampling operation was performed in the middle of each month. The harvested skin tissues were meticulously sectioned into pieces, immersed in RNA/DNA protector reagent (#9750, Takara, China), and then promptly transported on dry ice before being stored at −80 °C for subsequent utilization. It is imperative to note that all procedures involving animals adhered strictly to the Regulations of the State Science & Technology Commission of China for the ethical use of animals in scientific experiments, ensuring the highest standards of ethical conduct throughout the study.

### 2.2. Cell Lines and Cell Culture

The human embryonic kidney cell lines (HEK293T and HEK293A) were procured from the Chinese Academy of Sciences Cell Bank (Cell Bank, Chinese Academy of Sciences, Shanghai, China), while hair follicle dermal papilla cells (DPCs) were obtained from previous studies [19]. Cell cultures were maintained in high-glucose Dulbecco’s Modified Eagle Medium (DMEM) (Gibco, San Francisco, CA, USA) supplemented with Pyruvate, or in Dulbecco’s Modified Eagle Medium/Nutrient Mixture F-12 (DMEM/F12) (Gibco, CA, USA), with an additional 10% fetal bovine serum (BI, Herzliya, Israel), and a combination of penicillin and streptomycin (100 IU). The cells were incubated at 37 °C under 5% CO_2_.

### 2.3. Oligonucleotides, Plasmid Construct, and Adenovirus Transfection

Oligonucleotides were custom-synthesized by GenePharma (Shanghai, China). The specific sequence for the miR-144 mimic was designed as 5′-UACAGUAUAGAUGAUGUAC-3′. The precursor of miR-144 was cloned from the goat genome, and the obtained precursor sequence was then inserted into an adenovirus vector. High-concentration adenovirus viral fluid was obtained through the adenovirus packaging system following established protocols [19]. The 3′ untranslated region (UTR) of *Lhx2* was amplified and subsequently inserted into the psiCHECK-2 vectors (Promega, Madison, WI, USA) to generate the *Lhx2* plasmid variants (WT-*Lhx2*, MT-*Lhx2*-RoA, MT-*Lhx2*-RoB, and MT-*Lhx2*-RoC). The oligonucleotides and plasmids were introduced into cells through transfection using Lipofectamine 3000 reagent (Invitrogen, Carlsbad, CA, USA), following the manufacturer’s instructions. Additionally, Ad-miR-144 was introduced into DPCs using methods previously described in the literature [19].

### 2.4. RNA Extraction and Quantitative RT-PCR

Total RNA was extracted from tissues and cells employing the Eastep^®^ Super Total RNA Extraction Kit (Promega, Shanghai, China). First-strand cDNA was synthesized using the Thermo Scientific RevertAid First Strand cDNA Synthesis Kit (#K1622) (Thermo Scientific, San Francisco, CA, USA) according to the manufacturer’s instructions. The reagent employed for real-time fluorescence quantification was the SYBR Premix Ex Taq (Tli RNaseH Plus) kit (#RR420A, Takara, Beijing, China), and the instrument used was the Bio-Rad CFX96 Real-Time PCR Detection System. ACTB or U6 served as the internal control, and gene expression was determined using the 2^−ΔΔCt^ method. The primer details for this study are outlined in Table 1.

### 2.5. Western Blot Analysis

Western blot analysis was conducted to examine protein expression levels in cellular extracts. Total protein was isolated from cells using RIPA buffer (Beyotime, Shanghai, China), and its concentration was quantified with the BCA Protein Assay Kit (Beyotime, Shanghai, China). Subsequently, the proteins were separated by 12% SDS-PAGE and transferred onto PVDF membranes (Roche, Basel, Switzerland) using a vertical transfer system (Bio-rad, Hercules, CA, USA). After blocking the membranes with 5% skim milk overnight at 4 °C, they were probed with a primary antibody against Lhx2 (Bioss, Wuhan, China), diluted 1:100, for 2 h at room temperature. Following three PBS washes, the membranes were incubated with an HRP-conjugated secondary antibody (Bioss, Wuhan, China), diluted 1:1000, for 2 h. The membrane underwent four washes with PBST, with each wash lasting for 10 min. Placed on a chemiluminescence workstation, an appropriate amount of ECL developer (SuperSignal West Pico PLUS, Thermo Scientific, CA, USA) was added to cover the surface of the membrane, and images were captured using a WB imaging system (ChemiDoc XRS+, Bio-rad, CA, USA).

### 2.6. Verification the Sites of Target Gene

Target binding sites between miR-144-3p and Lhx2 were predicted using the TargetScan tool (http://www.targetscan.org/), and RNAhybrid (https://bibiserv.cebitec.uni-bielefeld.de/rnahybrid/), accessed on July 2016. The 3′ UTR of LHX2 was amplified from Shaanbei Cashmere goat skin cDNA, cloned into the psiCHECK2 vector (psiCHECK2-WT), and mutant constructs (psiCHECK2-MA, MB, MC) were subsequently generated according to the predicted binding sites using a one-step approach [20]. Lipofectamine 3000 (Invitrogen, CA, USA) was used to transfect the synthesized nucleotides and constructed reporter vectors into HEK293T cells. After 48 h, the activity of the firefly luciferase reporter gene was measured. Primer information is provided in Table 1. The specific transfection method and dual-luciferase assay details can be found in the Dual-Luciferase Reporter Gene Assay Kit (RG028, Beyotime, Shanghai, China).

### 2.7. Statistical Analysis

Statistical analysis was conducted using Student’s t-tests or one-way ANOVA to assess differences between groups. A significance level of *p* < 0.05 was considered statistically significant. Continuous variables, which were normally distributed, are presented as means ± standard deviation (SD). All experiments were independently performed in triplicate to ensure the reliability of the results.

## 3. Results

### 3.1. The Expression of Lhx2 and miR-144 in Goat Tissues

**Figure 1:**
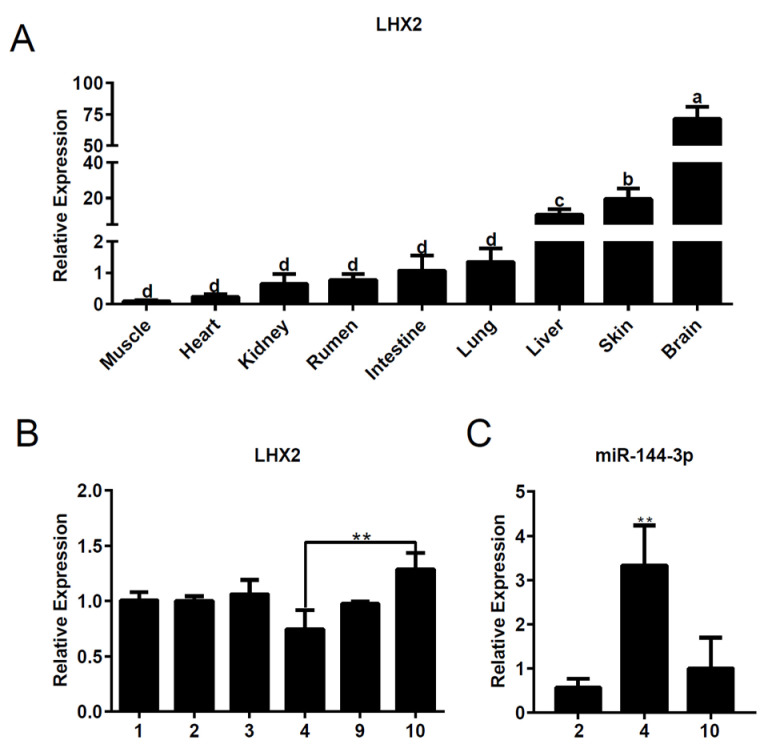
The expression of *Lhx2* and miR-144 in goat tissues. (**A**) The expression of *Lhx2* in nine different goat tissues. Different letters represent significant differences *p* < 0.05. (**B**) The relative expression of *Lhx2* in goat skin tissue at different times (1–2: catagen; 3–4: telogen; 9–10: anagen). (**C**) The relative expression of miR-144-3p in goat skin tissues at 2 (catagen), 4 (telogen) and 10 (anagen) months. ** represents *p* < 0.01.

Initially, we conducted an analysis of *Lhx2* expression across nine distinct tissues in June, including the skin tissue sampled over various months. The results indicated robust *Lhx2* expression in the liver, skin, and brain tissues (Figure 1A). Interestingly, the expression of *Lhx2* in the skin tissues displayed a time-dependent pattern, exhibiting a significant peak in October and decreased expression in April (Figure 1B). Simultaneously, miR-144-3p, identified as a potential regulator of *Lhx2* expression, exhibited elevated expression during the telogen phase and reduced expression during the catagen phase (Figure 1C).

### 3.2. miR-144-3p Targets Multiple Sites in Lhx2 3′ UTR

**Figure 2:**
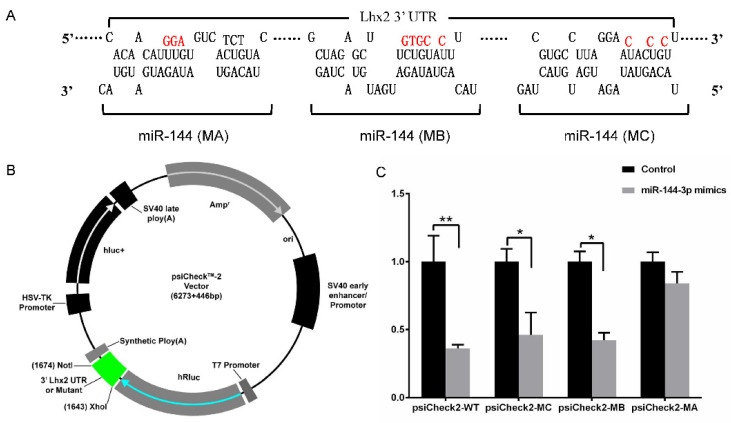
MiR-144-3p target sites in *Lhx2* 3′ UTR. (**A**) Three predicted target sites for miR-144 on *Lhx2* 3′ UTR fragment. The red bases indicate the mutant sites. (**B**) A sketch map of the 3′ UTR fragment of Lhx2 cloned into the psiCheckTM-2 vector. The green block is the wild or mutant fragment. (**C**) Dual Luciferase results. The control group contained synthetic oligonucleotides. psiCheck2-WT represents the wild type, psiCheck2-MA is the mutant with the three target sites for miR-144, psiCheck2-MB represents mutant MB and MC, and psiCheck2-MC represents the mutant (MC). * Represents *p* < 0.05, and ** represents *p* < 0.01.

Three potential target sites in the 3′ UTR of *Lhx2* for miR-144-3p were identified using the RNAhybrid tool and were marked as MA, MB, and MC (Figure 2A). The wild type and mutant type 3′ UTR fragments of *Lhx2* were cloned separately in the psiCheckTM-2 vector (Figure 2B). The dual luciferase results in significant downregulation of post-transcription *Lhx2* mRNA translation in psiCheck2-WT, psiCheck2-MC and psiCheck2-MB groups. However, no significant change was observed in the psiCheck2-MA group (Figure 2C). These results indicate that miR-144 strongly regulate post-transcriptional translation of *Lhx2*, and *Lhx2* 3′ UTR possessed at least three targets’ sites for miR-144-3p.

### 3.3. miR-144 Cloning into Adenoviruses and Validation

**Figure 3:**
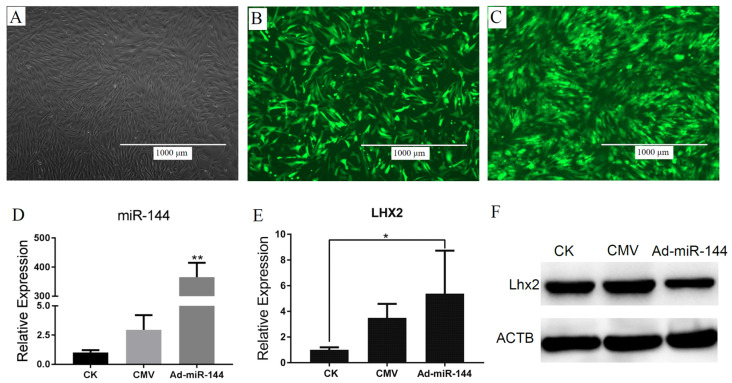
Validation of the targeting regulatory relationship to the Lhx2 gene by the adenoviruses system. (**A**) DPCs transfected with nothing for 48 h, CK group. (**B**) DPCs transfected with CMV for 48 h, CMV group. (**C**) DPCs transfected with Ad-miR-144 for 48 h, Ad-miR-144 group. (**D**) The expression of miR-144-3p in DPCs transfected with Ad-miR-144. (**E**) The expression of *Lhx2* in DPCs transfected with Ad-miR-144. (**F**) Western blot results for LHX2 protein. * Represents *p* < 0.05, ** represents *p* < 0.01.

Special primers targeting the miR-144 precursor were designed based on the Capra hircus sequence in the NCBI database (Capra hircus, chr19: 19580718-19580813 [-]), and were cloned into the pAdTrack-CMV vector. We successfully constructed the adenovirus expression vector for miR-144 and obtained a viral titer of over 4.8 × 10^9^ pfu/mL. DPCs were infected for 48 h with 100 MOI of adenovirus containing miR-144 (Figure 3C). Ad-miR-144 was effectively expressed in DPCs and was significantly higher in the treatment group than in the control groups (CK group and CMV group) (Figure 3D). Elevated levels of miR-144 led to a notable upregulation in the expression of *Lhx2* (Figure 3E). Concurrently, it was observed that the post-transcriptional translation of the Lhx2 gene was hindered (Figure 3F).

### 3.4. miR-144 Plays Its Biological Function by Targeting Lhx2 in DPCs

**Figure 4:**
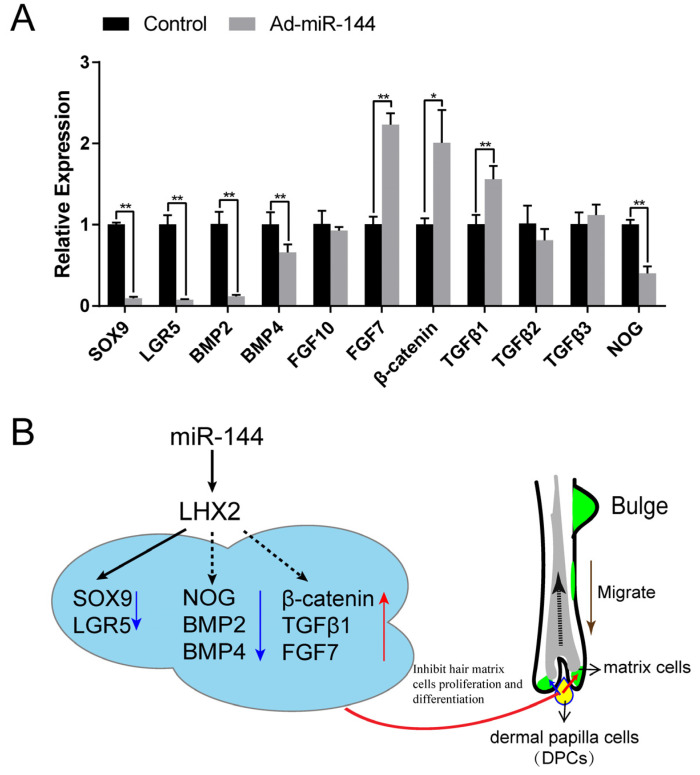
The effect of miR-144 on *Lhx2* expression in DPCs. (**A**) The relative expression of genes (*Lhx2*, *Sox9*, and *Lgr5* or *Bmp2*, *Bmp4*, *Fgf10*, Tgf*β*1 and *β-catenin*) on DPCs. (**B**) A sketch map for the function of miR-144 on the hair follicle. The continuous line indicates the direct effect, and the dotted line indicates the direct or indirect effect on hair follicle. * Represents *p* < 0.05, ** represents *p* < 0.01.

In order to delve deeper into the impact of miR-144 on DPCs, we conducted transfections with Ad-miR-144. Given the known targeting and regulation of Sox9 and Lgr5 by Lhx2, these two proteins were chosen as reference points. Our results revealed a noteworthy downregulation in the expression of *Sox9*, *Lgr5*, *Fgf10*, and *Bmp2*, while witnessing an upregulation in the levels of *Tgf*β1 and *β-catenin* (Figure 4A). To illustrate the relationship within the hair follicle, a schematic diagram was employed, visually portraying these interactions (Figure 4B). Combined with the expression characteristics of miR-144 during the telogen phase of the hair follicle cycle, it can be illustrated that miR-144 has an inhibitory effect on hair growth.

## 4. Discussion

The expression pattern of Lhx2 exhibits specificity concerning both tissue and temporal factors. Predominantly expressed in brain and skin tissues, Lhx2 displays variation corresponding to the hair follicle growth cycle in the skin [21]. This suggests the potential involvement of Lhx2 in the regression and regeneration of hair follicles. In line with these observations, our investigation revealed a time-specific expression pattern for miR-144, with higher levels observed during the telogen phase, aligning with previous findings [12]. Consequently, this prompted a more in-depth exploration into the intricate interplay between miR-144 and Lhx2.

Our investigation revealed that miR-144 exerts post-transcriptional regulation of Lhx2 expression by targeting a minimum of three sites within the 3′ UTR of the *Lhx2* gene. The overexpression of miR-144 led to an upregulation of *Lhx2* mRNA in DPCs, concomitantly causing a weakening of post-transcriptional translation of Lhx2. Consequently, a cascade of interconnected alterations ensued, resulting in modifications to the gene expression governed by Lhx2. Lgr5, a well-established target gene of the Wnt signaling pathway and a conserved marker of hair follicle stem cells [22,23], plays a crucial role in enhancing Wnt signaling and promoting cellular self-renewal [24]. Meanwhile, Lhx2 is known to maintain cellular environmental homeostasis [17,25,26]. Our investigations indicated that the expression of Lhx2 was inhibited, leading to the downregulation of Sox9 and Lgr5. Interestingly, our research findings align with previous studies, highlighting Lhx2’s differential regulation of Sox9, Tcf4, and Lgr5 in hair follicle stem cells, thereby influencing hair cycle development [27]. This experiment’s outcomes are in concordance with the aforementioned research, further reinforcing the understanding of the intricate regulatory network involving Lhx2 in the modulation of hair follicle development.

DPCs constitute the principal cells involved in hair follicle regeneration, growth, and apoptosis [2,28].

These multifaceted functions of DPCs are orchestrated through the secretion of various molecules. The heightened expression of miR-144 in DPCs led to the suppression of Bmp family members (Bmp2 and Bmp4), while concurrently enhancing the expression of β-catenin and the growth factor Fgf7. Notably, studies have elucidated that Bmp2 and Bmp4 act as inhibitors of hair follicle anagen induction by impeding the activation and expansion of epithelial stem/progenitor cells [29,30].

The reduction in Bmp2 and Bmp4 expression disrupts the induction of hair follicle anagen. Simultaneously, β-catenin, a pivotal component of the Wnt/β-Catenin pathway, plays a crucial role in regulating hair follicle morphogenesis and stem cell differentiation. It facilitates the differentiation of hair follicles into follicular keratinocytes in the skin [31,32]. FGF7, identified as a signaling factor from dermal papilla, actively contributes to the proliferation of hair germ cells and serves as a trigger for the initiation of a new hair cycle [33]. Within the hair bulb, the expression of noggin, an inhibitor of Bmp signaling known to impede hair growth, including vibrissae inhibition [34], underscores the robust antagonistic effect of noggin on Bmps expression. It is plausible that a simultaneous reduction in noggin and Bmps expression may serve to facilitate hair follicle regeneration. Although Tgfb1 play a pivotal role in regulating catagen expression [35], it is noteworthy that this process operates independently of transforming growth factor receptors [36].

## 5. Conclusions

In summary, our work unveils the novel and significant regulatory role of miR-144-3p in hair follicle development. It targets multiple sites on the *Lhx2* gene and influences key transcriptional factors and pivotal molecules, thereby controlling crucial processes in the hair matrix. This discovery not only enriches our understanding of the intricate molecular mechanisms underlying hair follicle growth but also paves the way for the development of novel therapeutic strategies targeting hair-related disorders. Moreover, it sets a foundation for further exploration of the crosstalk between miR-144-3p and other regulatory elements within the hair follicle microenvironment to uncover more comprehensive regulatory networks.

## Figures and Tables

**Table 1:** Primers used in this study.

Primer	Sequence (5′~3′)	Size (bp)
WT-*Lhx2*-F1	CCGCTCGAGTGACTCTCGGCCCCGCAC (Xho I)	424
WT-*Lhx2*-R1	ATAAGAATGCGGCCGCTACATTTTTGCTCTGGTC (Not I)	
MT-*Lhx2*-FoC	CTGCCACGTGCCTTAGGAACACCGCTTTATCTCCATACTTTGG	424
MT-*Lhx2*-RoC	CCAAAGTATGGAGATAAAGCGGTGTTCCTAAGGCACGTGGCAG	
MT-*Lhx2*-FoB	AGCTTCGTGCTCTTCAAAGACTGCCACGTGCCTTAG	424
MT-*Lhx2*-RoB	CTTTGAAGAGCACGAAGCTCTAGCCAACTTAAATTA	
MT-*Lhx2*-FoA	ACATGGATGTCATCTTACAGTTTTGTGGACTGAGC	424
MT-*Lhx2*-RoA	GTAAGATGACATCCATGTTGTGTTGCAGATAGATC	
qmiR-144-RT	GTCGTATCCAGTGCAGGGTCCGAGGTATTCGCACTGGATACGACTACATC	
qmiR-144-F	TGGCGGTACAGTATAGATG	
qmiR-144-R	GTGCAGGGTCCGAGGT	
U6-F	CTCGCTTCGGCAGCACA	94-
U6-R	AACGCTTCACGAATTTGCGT	
ACTB-F	TCTGGCACCACACCTTCTAC	102
ACTB-R	TCTTCTCACGGTTGGCCTTG	
*Lhx2*-qF	CAACCCTCTGGGTCTTCCCTACT	186
*Lhx2*-qR	CGCTTCGTCTTCTGGCTGCTC	
*Lgr5*-qF1	AGCTTGGTGGTTCTAGGATTTCA	174
*Lgr5*-qR1	GGCGCCATTCAAAGTCAGTG	
*Sox9*-qF1	GCCCAACGCCATCTTCAAGG	294
*Sox9*-qR1	TACTGGTCGAACTCGTGGAC	
*β-catenin*-qF1	TATTGGTGCCCAGGGAGAAC	297
*β-catenin*-qR1	ACAGGCCAATCACAATGCAAG	
*Fgf7*-qF1	AAGTTGCACAGGGCAGACAA	94
*Fgf7*-qR1	GTTGCTGAGATGCTGTTTGCT	
*Fgf10*-qF1	GGAAAGGTCAGCGGTACCAA	244
*Fgf10*-qR1	ACATTTGCCTCCCATTGTGC	
*Bmp4*-qF1	TCCCCAAAGCCTGTTGTGTT	121
*Bmp4*-qR1	CGGCAACCACATCCCTCTAC	
*Bmp2*-F1	CTACATGCTGGACTTGTACC	232
*Bmp2*-R1	CCGAAAGACCTGAAGTTCTG	
*Tgfβ3*-qF1	ACAGTGATGATGATCCGGGC	203
*Tgfβ3*-qR1	GTCAATGTAGAGAGGGCGCA	
*Tgfβ2*-qF1	GCGCTACATCGACAGCAAAG	82
*Tgfβ2*-qR1	TTCGTGAACAGCATCGGTGA	
*Tgfβ1*-F1	CTAGCTCGCACAGCATATAC	277
*Tgfβ1*-R1	CGAAAGCCCTCTATTTCCTC	
*Nog*-qF1	CACTATCTCCACATCCGCCC	153
*Nog*-qR1	CATGAAACCCGGGTCGTAGT	

## Data Availability

The original contributions presented in the study are included in the article, further inquiries can be directed to the corresponding author.
